# Nanomaterials-Mediated Therapeutics and Diagnosis Strategies for Myocardial Infarction

**DOI:** 10.3389/fchem.2022.943009

**Published:** 2022-07-07

**Authors:** Qingbo Lv, Boxuan Ma, Wujiao Li, Guosheng Fu, Xiaoyu Wang, Yun Xiao

**Affiliations:** ^1^ Key Laboratory of Cardiovascular Intervention and Regenerative Medicine of Zhejiang Province, Sir Run Run Shaw Hospital, Zhejiang University School of Medicine, Hangzhou, China; ^2^ Department of Cardiology, Sir Run Run Shaw Hospital, Zhejiang University School of Medicine, Hangzhou, China; ^3^ Qiushi Academy for Advanced Studies, Zhejiang University, Hangzhou, China

**Keywords:** myocardial infarction, nanomaterials, targeted delivery, macrophage, diagnosis

## Abstract

The alarming mortality and morbidity rate of myocardial infarction (MI) is becoming an important impetus in the development of early diagnosis and appropriate therapeutic approaches, which are critical for saving patients’ lives and improving post-infarction prognosis. Despite several advances that have been made in the treatment of MI, current strategies are still far from satisfactory. Nanomaterials devote considerable contribution to tackling the drawbacks of conventional therapy of MI by improving the homeostasis in the cardiac microenvironment via targeting, immune modulation, and repairment. This review emphasizes the strategies of nanomaterials-based MI treatment, including cardiac targeting drug delivery, immune-modulation strategy, antioxidants and antiapoptosis strategy, nanomaterials-mediated stem cell therapy, and cardiac tissue engineering. Furthermore, nanomaterials-based diagnosis strategies for MI was presented in term of nanomaterials-based immunoassay and nano-enhanced cardiac imaging. Taken together, although nanomaterials-based strategies for the therapeutics and diagnosis of MI are both promising and challenging, such a strategy still explores the immense potential in the development of the next generation of MI treatment.

## 1 Introduction

The growing burden of cardiovascular disease (CVD) is emerging as a major public health issue, particularly in the context of the COVID-19 pandemic, thus presenting an alarming risk to the healthcare settings worldwide (Zhao et al., 2019; Hessami et al., 2021). Myocardial infarction (MI), the most severe type of CVD, is caused by the rupture of an atherosclerotic plaque, leading to irreversible necrosis of cardiac tissue (Ong et al., 2018; Musher et al., 2019). The occurrence of MI can result in insufficient myocardial oxygen supply, which causes the necrosis and apoptosis of cardiomyocytes and a large area of myocardium tissue damage. The damaged areas of myocardial tissue are incapable of effective regeneration and restoration due to the insufficient proliferation capacity of cardiomyocytes (Peet et al., 2020; Richards et al., 2020). Since the mortality and morbidity rates are comparatively higher in MI, early diagnosis and proper therapeutic measures are critical for saving patients’ lives. However, diagnosis and appropriate therapeutic approaches to MI remain the main challenge due to the complexity of their pathophysiology conditions (Tibaut et al., 2017).

Currently, timely revascularization after MI, including percutaneous coronary intervention (PCI) (Doenst et al., 2019; Neumann et al., 2019), thrombolytic treatment (McDonagh et al., 2021), and bypass surgery (Doenst et al., 2019), is critical to improving cardiac function and preventing post-infarction pathophysiological remodeling. While it is widely recognized that the current therapeutic means remain limited since reperfusion of coronary arteries paradoxically induces cardiomyocyte death, known as myocardial ischemia-reperfusion injury (MRI) (Jin et al., 2021; Lin et al., 2022). Our research group has conducted extensive research on the pathogenesis of MI and MRI and found several promising therapeutic targets (Minamino et al., 2010; Yang et al., 2017; Bi et al., 2018). Besides, pharmacotherapy, including anti-platelet, anti-arrhythmic drugs, and angiotensin-converting enzyme inhibitors, has also been shown to be limited in minimizing the infarct size and improving prognosis, owing to non-targeted drug distribution and side effects, and short half-life of some drugs (Reed et al., 2017; Li et al., 2021; Luan et al., 2021). In this regard, it is imperative to look for an effective, alternative, and exact method for the timely diagnosis of MI and effective treatment of MI.

**FIGURE 1:**
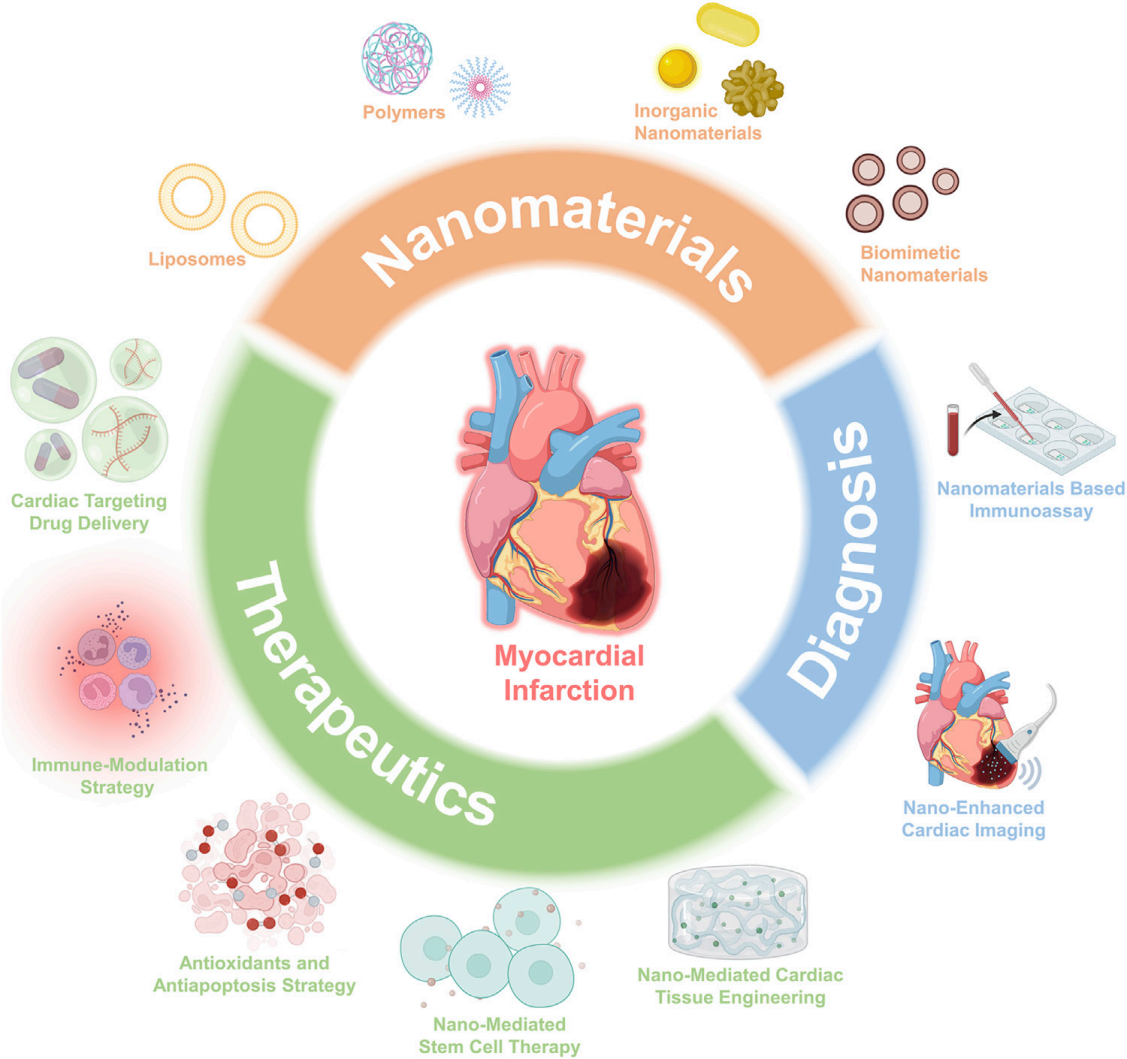
Schematic overview of the advances of nanomaterials and applications for both therapeutics and diagnosis.

Recent advances in nanotechnology have opened up new avenues for biomedicine and healthcare settings, which have been widely applied to design unique diagnostic and therapeutic platforms that can overcome the defects of current techniques (Son et al., 2014; Donahue et al., 2019; Hu et al., 2020). Nanomaterials with intrinsic physicochemical properties, such as the high surface area to volume ratio, high surface energy, tunability, and ease of surface property modification (Blanco et al., 2015; Shi et al., 2017), could influence drug delivery through vascular systems within the cardiovascular system (Wang et al., 2022a), which suggests a growing potential for the clinical diagnosis and therapy of cardiology (Matoba and Egashira, 2014; Cheraghi et al., 2017). Besides, nanomaterials that serve as platforms for loading multiple functional groups and integrating surface moieties can selectively direct the cell-specific activities such as anti-inflammation, antiapoptosis, and immune modulation (Matoba and Egashira, 2014), which is very useful in regulating the functions of targeted cells, providing exciting opportunities for potential cardiovascular applications. Of note, by integrating nanomaterials with unique optical and photothermal conversion properties, the functions of therapy and diagnosis can be combined in a single platform, enabling the accurate diagnosis and monitoring of the treatment. This review article provides a comprehensive description of nanomaterial’s applications in the therapeutics and diagnosis of MI, intending to bridge the needs in MI treatments with the innovations of nanomaterials (Figure 1).

## 2 Nanomaterials-Mediated Therapeutics Strategies for MI

### 2.1 Cardiac Targeting Drug Delivery

At present, pharmaceutical is one of the most important clinical standards of care for patients in the acute phase of MI. Several studies have attempted to apply small molecular active drugs and other biologics such as miRNAs, therapeutic transgenes, siRNAs, etc., for the MI therapeutics (Somasuntharam et al., 2013; Gao et al., 2019). Although these therapeutic agents display protective roles in improving cardiac functions and inhibiting cardiac remodeling to a certain extent, their clinical efficacy is hindered due to their poor solubility, off-target effects, and short circulation times (Fan et al., 2020). To overcome these obstacles, drugs and biologics can be loaded into various nanocarriers, which have become an effective way to reduce off-target effects and increase cycle times compared with freely administered small molecules and biologics (Ding et al., 2022a). In this part, we classify nanomaterials into liposomes, polymers, inorganic materials, and biomimetic nanomaterials, and review their research progress as drug carriers for MI therapy. The summary of the latest studies on the use of these four kinds of nanomaterials for the treatment of MI is provided in Table 1.

**TABLE 1:** Summary of the nanomaterials used for the therapy of MI.

Category	Nanomaterials	Therapeutic agent	Administration route	Model	Results	References
Liposomes	Liposomes	PARP-1 inhibitor	Intravenous administration	Myocardial I/R injury	9-fold and 1.5-fold higher efficiencies of PARP-1 inhibition in cardiomyocytes and macrophages, respectively	Dasa et al. (2015)
Liposomes	Liposomes	AMO-1	Intravenous administration	MI	Relieved ischemic arrhythmia by silencing of miR-1 and restored the depolarized resting membrane potential	Liu et al. (2014)
Liposomes	Liposomes	Berberine	Intravenous administration	MI	Preserved the cardiac ejection fraction at day 28 after MI	Allijn et al. (2017)
Liposomes	SLNs	TFDM	Oral delivery	Myocardial I/R injury	Decrease infarct area, cardiac enzyme, and inflammatory factors	Tan et al. (2017)
Liposomes	Liposomes	MI antigens and rapamycin	Intradermal injection	MI	Attenuated inflammation in the myocardium, inhibited adverse cardiac remodeling, and improved cardiac function	Kwon et al. (2021)
Polymers	PLGA	AdSCs and simvastatin	Intravenous administration	MI	Contributed to significant cardiac functional recovery with intrinsic myocardial tissue regeneration	Yokoyama et al. (2019)
Polymers	PLGA	TLR4 inhibitor TAK242	Intravenous administration	Myocardial I/R injury	Reduced the infarct size by inhibiting recruitment of Ly-6C^high^ monocytes to the heart, and decreased circulating HMGB1, and NF-κB activation and cytokine expressions	Fujiwara et al. (2019)
Polymers	PLGA	Irbesartan	Intravenous administration	Myocardial I/R injury	Inhibitd the recruitment of inflammatory monocytes to the IR heart, reduced the infarct size, and ameliorated left ventricular remodeling	Nakano et al. (2016)
Polymers	PEG-PLA	miR-133	Intravenous administration	MI	Improvd the cardiac function, reduced the myocardial infarction area, and inhibited cardiomyocyte apoptosis, inflammation, and oxidative stress	Sun et al. (2020)
Polymers	Chitosan and alginate	PGF	Intramyocardial injection	MI	Increased left-ventricular function, vascular density, and serum anti-inflammatory cytokine levels, and decreased scar area formation and serum pro-inflammatory cytokines levels	Binsalamah et al. (2011)
Polymers	PEG-DGL	miR-1 inhibitor	Intravenous administration	MI	Decreased apoptotic cell death in the infarct border zone and reduced myocardial infarct size	Xue et al. (2018)
Polymers	PGEA	miR-499 and pVEGF	Intravenous administration	MI	Restored heart function and suppressed cardiac hypertrophy	Nie et al. (2018)
Polymers	PLGA	IGF-1	Intramyocardial injection	MI	Prevented cardiomyocyte apoptosis, reduced infarct size, and improved left ventricle ejection fraction	Chang et al. (2013)
Polymers and Inorganic Nanomaterials	Fe_3_O_4_, silica-PEG	CD63 and MLC antibodies	Intravenous administration	MI	Reduced infarct size and improved left-ventricle ejection fraction and angiogenesis	Liu et al. (2020)
Inorganic Nanomaterials	Iron	CD45 and MLC antibodies	Intravenous administration	Myocardial I/R injury	Reduced scar formation and improved pump function of the hearts	Cheng et al. (2014)
Inorganic Nanomaterials	Gold	DNAzyme functionalized gold nanoparticles	Intramyocardial injection	MI	Resulted in significant anti-inflammatory effects and improvement in acute cardiac function	Somasuntharam et al. (2016)
Biomimetic Nanomaterials	Exosomes	hiPSCs and hiPSCs-derived exosomes	Intramyocardial injection	MI	Increased cardiac function, reduced scar size and cell apoptosis, and promoted angiogenesis	Gao et al. (2020)
Biomimetic Nanomaterials	Monocyte mimics	MSC-derived EVs	Intravenous administration	Myocardial I/R injury	Promoted endothelial maturation during angiogenesis and modulated macrophage subpopulations	Zhang et al. (2020)
Biomimetic Nanomaterials	EVs	miR-21	Intramyocardial injection	MI	Inhibited cell apoptosis and led to significant cardiac function improvement	Song et al. (2019)
Biomimetic Nanomaterials	IONPs	Exosome-mimetic extracellular NVs	Intramyocardial injection	MI	Induced an early shift from the inflammation phase to the reparative phase, reduced apoptosis and fibrosis, and enhanced angiogenesis and cardiac function recovery	Lee et al. (2020)
Polymers and Biomimetic Nanomaterials	MIONs and PLA-PCB	PS	Intravenous administration	MI	Preserved the left ventricular remodeling and improved the cardiac function, and realized accurate diagnosis and site-specific treatment of the inflammatory stage	Chen et al. (2017)

PARP-1, poly (ADP-ribose) polymerase 1; I/R, ischemia–reperfusion; AMO-1, anti-miR-1, antisense oligonucleotides; MI, myocardial infarction; SLNs, solid lipid nanoparticles; TFDM, total flavonoid extract from dracocephalum moldavica L; PLGA, Poly (lactic-co-glycolic acid); AdSCs, adipose-derived stem cells; TLR4, toll-like receptor 4; HMGB1, high mobility group box 1; group box 1; PEG, polyethylene glycol; PLA, poly (lactide); PGF, placental growth factor; DGL, dendrigraft poly-L-lysine; PGEA, poly(glycidyl meth-acrylate); pVEGF, plasmid encoding vascular endothelial growth factor; IGF-1, insulin-like growth factor-1; MLC, myosin light chain; hiPSCs, human induced pluripotent stem cells; MSC, mesenchymal stem cell; EVs, extracellular vesicles; IONPs, Iron oxide nanoparticles; NVs, nanovesicles; MIONs, magnetic iron oxide nanocubes; PCB, polycarboxybetaine; PS, phosphatidylserine.

#### 2.1.1 Liposomes

Liposomes are recognized as artificial vesicles consisting of an aqueous core surrounded by a bilayer phospholipid membrane, which is usually amphiphilic with an aqueous interior. Due to their unique amphiphilic properties, liposomes have been used as nanocarriers to encapsulate both hydrophilic and hydrophobic pharmaceutical agents (Torchilin, 2005). Encapsulation of drugs into liposomes can protect and controls their release and reduce systemic toxicity by minimizing dose requirements (McCarthy, 2010; Singh et al., 2016). As known, delivery of drugs to the target site is particularly difficult on account of high shear stresses in the cardiovascular system, limiting the clinical utility of drugs. Recently, many groups have successfully structured target-specific liposomes for delivering therapeutic agents and micro-RNA via modifications of different targeting ligands, including antibodies, antibody fragments, and peptides to interface liposomes. Dasa et al*.* constructed cardiomyocyte-targeted liposome vectors for delivering the poly (ADP-ribose) polymerase 1 (PARP-1) inhibitor (AZ7379) by adding cardiomyocyte-specific I-1 peptide to the formulation (Dasa et al., 2015). Such a cardiomyocyte-targeted liposome approach increased the availability of AZ7379 in the infarct/border zone at 24 h post-injection as compared to free AZ7379. The presence of total AZ7379 in circulation was prolonged when it was formulated as a liposomal, resulting in a 3-fold improvement in PARP-1 inhibition efficiency when evaluating all cell types using I-1 liposomes. In another case, Liu et al*.* explored anti-cardiac troponin I (cTnI) antibody modified liposomes to deliver anti-miR-1 antisense oligonucleotides (AMO-1) to ischemic myocardium tissues to increase the therapeutic efficiency and inhibit off-target effects of AMO-1 (Liu et al., 2014). *In vivo* imaging results revealed that cTnI-liposomes (cT-LIP) significantly promoted the accumulation of fluorescent trackers in the ischemic foci compared with liposomes (LIP). The liposome vector can deliver AMO-1 to the ischemic myocardium of MI rats, further verifying the effect of AMO-1 on alleviating ischemic arrhythmia by silencing miR-1 in the ischemic myocardium.

However, liposomes are quickly cleared by the liver and the reticuloendothelial system, so they are often coated with biocompatible polymers (such as PEG) to improve blood circulation time and increase their stability in the blood. Allijn et al*.* constructed a liposomal nanocarrier prepared by ethanol injection method for site delivery of berberine in MI (Allijn et al., 2017). MI leads to poor cardiac remodeling and cardiac dysfunction, including increased end-diastolic and end-systolic volumes, whereas berberine-loaded liposomes can effectively reduce poor cardiac remodeling by prolonging circulation, thus alleviating cardiac dysfunction in mice with MI. Compared with control liposomes and free berberine liposomes, berberine liposomes significantly preserved cardiac ejection fraction at 28 days after MI by 64%. This result proved that the liposome-based nanomaterials significantly improved the treatment effectiveness of the drugs for MI.

Since phospholipids are easily oxidized and degraded, the stability of liposomes remains a problem that needs to be considered in their applications (Kahraman et al., 2017). In this regard, lipid nanoparticles based on the solid matrix have emerged as potential drug carriers, which are defined as solid lipid nanoparticles (SLNs). These nanoparticles are usually prepared from biodegradable, biocompatible, and physiological lipids, so the toxicity problems associated with polymer nanoparticles can be minimized (Müller et al., 2002; Paliwal et al., 2020). The core of SLN consists of solid lipids with diameters of approximately 50–1,000 nm. The nanosizes of SLN favor their accumulations in the ischemic myocardium. Tan et al*.* loaded SLN with Total Flavonoid Extract of *Dracocephalum Moldavia L.* (TFDM) to form TFDM-loaded solid lipid nanoparticles (TFDM-SLNs) (Tan et al., 2017). With the help of SLNs, oral delivery of TFDM-SLNs showed better protective effects for the myocardial ischemia-reperfusion injury than TFDM alone.

#### 2.1.2 Polymers

Polymer structures consisting of two or more blocks with different hydrophobicity have been widely used as drug carriers. Due to the structure of the hydrophilic shell with a hydrophobic core, polymers can host water-insoluble drugs, while the hydrophilic corona can be additionally modified for the attachment of water-soluble drugs or targeting moieties. To date, several structures, such as hydrogels, network-like scaffolds, microparticles, nanospheres, and nano-shells, have been formed by polymers for drug delivery (Amin et al., 2020). They can achieve multiple tasks by regulating their hydrophobicity, degradability, and potential reabsorption. The extensive modifiable end groups of polymers enable the improvement of their performance and reduction of adverse drug reactions in medical applications (Won et al., 2013).

Poly (lactic-co-glycolic acid) (PLGA), an FDA-approved material, is a copolymer that has been widely used for medical applications due to its low toxicity, high biocompatibility, and biodegradability (Rocha et al., 2022). In this route, many PLGA-based nano delivery systems have been used to deliver various small molecule drugs, such as growth factors, bioactive peptides, receptor agonists, or inhibitors (Yokoyama et al., 2019, Ma Q et al., 2018). For example, Fujiwara et al*.* constructed PLGA nanoparticles encapsulated with toll-like receptor 4 (TLR4) inhibitor TAK242 to inhibit myocardial ischemia-reperfusion (I/R) injury in mice (Fujiwara et al., 2019). With the help of PLGA carriers, TAK212 was transported and recruited in the damaged myocardium and inhibited Ly6c-overexpressed monocytes, thereby avoiding overactivated inflammation. Nakano et al*.* developed bioabsorbable nanoparticles incorporating irbesartan, an angiotensin II type 1 receptor blocker with a peroxisome proliferator-activated receptor (PPAR) γ agonistic effect, to inhibit the recruitment of monocytes (Nakano et al., 2016). The irbesartan nanoparticles decreased the infarct size and alleviated the cardiac remodeling after cardiac I/R injury. Similarly, PEG is a highly water-soluble, non-conductive flexible polymer with good biocompatibility (Ding et al., 2022b; Lu et al., 2022). It can effectively deliver various therapeutic drugs or small molecules by promoting immune escape and spatial rejection (Shao et al., 2017). Sun et al*.* designed PEG-PLA nanoparticles coated with the arginine-glycine-aspartic acid tripeptide (RGD) for the delivery of miR-133 (Sun et al., 2020). In this study, the PEG acted as the connector for the PLA and RGD. The modification of PEG and RGD promoted the distributions of these nanoparticles in the infarcted hearts, hence inhibiting the cardiomyocyte apoptosis, inflammation, and oxidative stress via the protective effects of miR-133.

As natural polymer materials, polysaccharides, including chitosan, dextran, alginate, and pectin, have the advantages of safe, degradable, uniform, and hydrophilic properties. Polysaccharides have received close attention in the therapeutic applications of cardiovascular diseases due to the extensive reaction groups distributed, such as amino, hydroxyl, and carboxyl groups, which enable the researchers to synthesize various polysaccharide derivatives for different purposes. Numerous studies have developed polysaccharides and their derivatives as drug delivery carriers for MI treatment. Binsalamah et al*.* constructed a novel chitosan-alginate composite nanoparticles loaded with placental growth factor (PGF), which could provide a sustained slow-release of PGF and enhance the positive effects of the growth factor in the setting of acute myocardial ischemia (Binsalamah et al., 2011). Besides, Luo et al*.* reported that chitosan-acetylsalicylic acid (ASA) nanoparticles slowly released ASA in a pH-dependent way to exert its antithrombotic effect (Luo et al., 2018).

#### 2.1.3 Inorganic Nanomaterials

**FIGURE 2:**
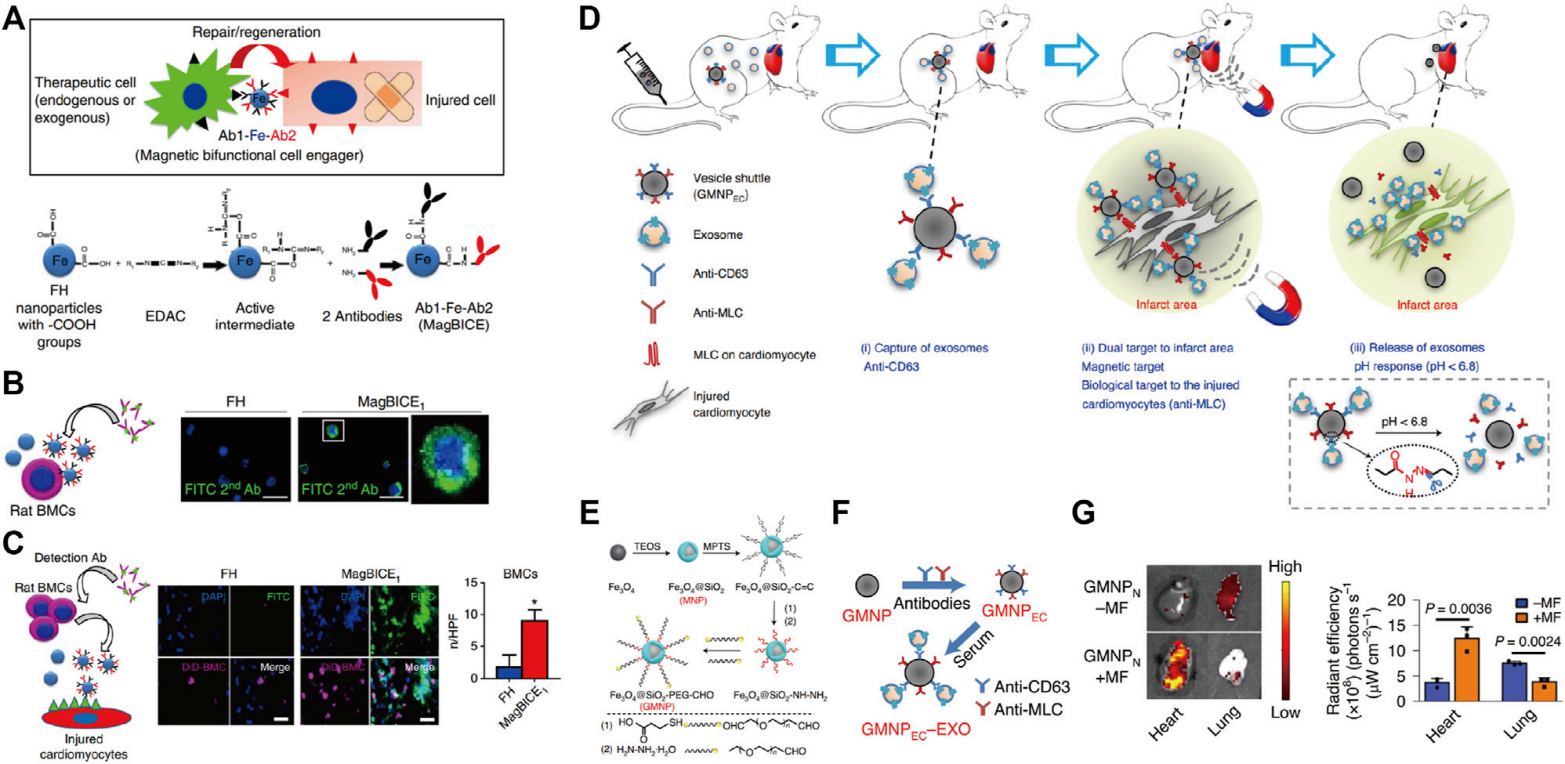
Applying antibody-conjugated magnetic nanoparticles for the targeting delivery in the treatment of MI. Magnetic antibody-linked nanomatchmakers for therapeutic stem cell targeting in the treatment of MI. **(A)** Schematic representations of the cell matchmaking by magnetic bifunctional cell engager 1 (MagBICE) and the preparation of MagBICE nanoparticles. **(B)** Fluorescent microscopic images showing the binding of MagBICE_1_, but not unconjugated Feraheme (FH), to rat bone marrow mononuclear cells (BMCs). **(C)** Fluorescent microscopic images showing MagBICE_1_ conjoined BMCs (DiD-labelled, magenta) with injured cardiomyocytes, Adapted with permission from Cheng et al., 2014. **(D)** Schematic representations of vesicle shuttle which can effectively collect, transport, and release circulating exosomes to infarcted areas of the hearts by the core-shell-corona structure by applying antibody-conjugated magnetic nanoparticles. **(E)** Schematic of the GMNP fabrication. **(F)** Schematic of the construction of endothelial cell denoted surface-grafted magnetic nanoparticles (GMNP_EC_) by the addition of anti-CD63 and anti-myosin light chain (MLC) antibodies to GMNPs, and the attachment of rat-derived exosomes from the *in vitro* serum to the anti-CD63 on the surface of GMNP_EC_ nanoparticles (GMNP_EC_–EXO). **(G)** *Ex vivo* fluorescent imaging of intravenously (*i.v.*) injected RhB-labelled GMNPN in MI-model rats with or without subsequent exposure to an external magnetic field. Adapted with permission from Liu et al., 2020.

Due to the unique physical, electrical, magnetic, and optical properties of the nanomaterial itself, many inorganic materials, such as gold, iron, and silica, have been used to synthesize nanostructured materials for drug delivery (Wang et al., 2022b). Inorganic nanoparticles are ideal for cardiac targeting drug delivery on account of their wide availability, rich functionality, good biocompatibility, potential targeted delivery, and controlled release of carried drugs (Sperling and Parak, 2010). More importantly, they are reported to regulate the macrophage phenotype modulation (Chenthamara et al., 2019; Zhao et al., 2021). Iron oxide nanoparticles are the most common inorganic nanomaterials with good biocompatibility and low toxicity (Arias et al., 2018). Excellent paramagnetism enables ferric oxide nanoparticles to deliver drugs, exosomes, and stem cells in a directional manner through magnetic fields, which are also widely used in the cardiovascular field (Ottersbach et al., 2018). Cheng et al*.* designed iron nanoparticles conjugated with two antibodies (one against antigens on therapeutic cells and the other directed at injured cells) to produce the magnetic bifunctional cell engager (MagBICE) (Figure 2A) (Cheng et al., 2014). The MagBICE showed great capacities for binding specifically to the stem cells and injured cardiomyocytes, and conjoining the two (Figure 2B). When it was delivered intravenously to the rats post-MI, the nanoparticles could be enriched in the injured myocardium mediated by the iron core of nanoparticles under the guidance of external magnetic fields (Figure 2C). Then the conjugated antibodies at the surfaces of nanoparticles mediated the interaction between therapeutic exogenous bone marrow-derived stem cells and injured cardiomyocytes, thus inhibiting the adverse cardiac remodeling and promoting the recovery of the cardiac functions. Similarly, Liu et al*.* designed antibody-conjugated magnetic nanoparticles with Fe_3_O_4_ as the core and PEG-modified silica as the shell (Figures 2D,E) (Liu et al., 2020). The PEG was conjugated with two types of antibodies that bind extracellular vesicles or injured cardiomyocytes (Figure 2F). Under the effect of antibodies and guidance of magnetic fields, exosomes could be recruited from the peripheral circulation to infarcted myocardium and exert its therapeutic effects (Figure 2G).

Silicon nanoparticles are another commonly used inorganic nanomaterial (O'Farrell et al., 2006). Porous silicon (PSi) has a unique chemical surface and biodegradability, which is beneficial in controlling drug release. Ferreira et al*.* synthesized PSi nanoparticles loaded with atrial brain natriuretic peptide (ANP) to target specific ANP receptors in cardiomyocytes and cardiac fibroblasts (Ferreira et al., 2017). Synthetic nanoparticles can accumulate in infarcted hearts of rats by intramyocardial injection, especially in the intima of the ischemic region. In another study, PSi nanoparticles loaded with Wnt3a protein, a cell-penetrating peptide, improved transplantation survival of mesenchymal stem cells (MSCs) and increased their antioxidant stress activity (Qi et al., 2019).

Gold nanoparticles, with the good properties of easy preparation and surface functionalization, are also widely applied for the delivery of bioactive substances such as drugs, proteins, and enzymes. In addition to their advantage of good conductivity in cardiac repair, they can also benefit the infarcted hearts due to their antioxidant and anti-inflammatory abilities, mediated by the binding of free radicals and inhibiting the tumor necrosis factor-α (TNF-α) expressions, respectively (de Carvalho et al., 2018). More importantly, gold nanoparticles themselves show a good capacity for accumulation in the heart during MI to decrease the infarction size and inhibit fibrosis (Tian et al., 2018). Besides, the cationic gold nanoparticles are also welcomed to use as the gene carriers due to their efficient interactions with DNA and negatively charged plasma membrane (Ghosh et al., 2008). Somasuntharam et al*.* developed the deoxyribozyme (DNAzyme) functionalized gold nanoparticles to catalytically silence TNF-α (Somasuntharam et al., 2016). The spherical nanostructure of gold nanoparticles facilitated the internalization of DNAzyme into macrophages to selectively knock down TNF-α, inhibiting the inflammatory responses during the acute stage of MI eventually. Similarly, gold nanoparticles incorporated with transcription factors can also mediate the effective gene reprogramming therapy *in vivo*. Chang et al*.* designed the cationic gold nanoparticles loaded with transcription factors of Gata 4, Mef2c, and Tbx5 for cardiac reprogramming in MI (Chang et al., 2019). Under the mediation of gold nanoparticles, these nano-complexes showed high delivery efficiency of the transcription factors into the fibroblasts and promoted their reprogramming into cardiomyocytes, thus achieving cardiac regeneration during MI.

#### 2.1.4 Biomimetic Nanomaterials

Applying biomimetic strategies to design nanocarriers has extensively promoted the application of nanomaterials for drug delivery. Bioinspiration and biomimicry technologies can not only simulate biological materials by their chemical structure but also by their biological functions. By mimicking the cell membranes, biomimetic nanomaterials can delay the clearance of MPS and increase circulation time (Guo et al., 2022). Leukocytes are one of the most crucial immune components of the immune system. They can trigger the aggregations of intercellular adhesion molecules in the inflammatory endothelial cells and then penetrate the endothelial layer to reach the diseased tissue (Palomba et al., 2016). Inspired by this, Zhang et al*.* developed mesenchymal stem cells (MSCs)-derived exosomes coated by monocyte membrane, which showed the characteristics of the recruitment feature of monocyte to I/R lesions. As such, these exosomes could target the injured area to provide a therapeutic effect (Zhang et al., 2020).

Extracellular vesicles (EVs) are another common type of biomimetic nanomaterials. As novel endogenous biological nanoparticles, EVs showed stable physical and chemical properties and excellent histocompatibility (Cai et al., 2021). EVs can participate in transmitting various proteins, lipids, nucleic acids, or other active substances between cells and have the ability to home to target tissues or cells (Walters et al., 2019; Chen et al., 2020). Notably, the endogenous immune cells can secrete some circulating EVs for cardiac protection (Xiong et al., 2021), although the therapeutic effects of natural EVs remain limited due to the low purity and weak targeting (Villa et al., 2019). So EVs could be good candidates as biomimetic nanomaterials for drug delivery as well as a practical approach of cell-free strategy for the treatment of ischemic heart disease (Xiong et al., 2021). In order to solve the shortage of natural EVs, many methods have been developed to control their biological distributions so that they can fully improve their therapeutic effect, reduce the area of MI, promote angiogenesis, and restore cardiac function (Adamiak and Sahoo, 2018). Song et al*.* transfected miR-21 plasmid into the human embryonic kidney cell line to produce miR-21-rich EVs (miR-21-EVs) (Song et al., 2019). *In vivo* and *in vitro* experiments show that miR-21-EVs could significantly promote the expression of miR-21. It inhibited cardiomyocyte apoptosis and improved cardiac function by reducing the expression of programmed cell death protein 4 (PDCD 4), a target gene of miR-21. Besides, stem cells derived EVs can also be used as an effective material for biomimetic nanomaterials. Lee et al*.* cultured MSC with iron oxide nanoparticles (IONPs) to form IONPs incorporated MSCs (IONP-MSCs) (Lee et al., 2020). Then they obtained the exosome-mimetic extracellular nanovesicles (NVs) containing IONPs by serial extrusion of IONP-MSCs. They not only have similar function and size to the MSC-derived exosomes but also are magnetic. Through intramyocardial injection and magnetic guidance, IONP-NVs successfully promoted the early transfer from the inflammation stage to the reparative stage of MI, resulting in significantly alleviated apoptosis and fibrosis.

### 2.2 Immune-Modulation Strategy

The occurrence of cardiac ischemic injury leads to the apoptosis of cardiomyocytes, which triggers an increase in reactive oxygen species (ROS) and damage-associated molecular pattern (DAMP), promoting the recruitment of a large number of neutrophils, monocytes/macrophages, and lymphocytes from the circulation to the infarcted areas. Normally, these inflammatory cells help maintain the immune homeostasis by removing damaged cells and recovering myocardial tissue. However, severe cardiac damage with excessive inflammation can aggravate tissue damage and impede cardiac repair, eventually leading to heart failure (Haider et al., 2019). Among cardiac immune cells presented in MI, resident macrophages of the heart are the most critical types for modulating the inflammatory responses. Macrophages in the infarcted areas are pro-inflammatory M1 type in the early stage and gradually differentiate into reparative M2 type in the later stage (Ma Y et al., 2018). So, the strategies that can modulate the shift of the macrophage phenotypes have become attractive approaches for preventing myocardial injury after MI.

**FIGURE 3:**
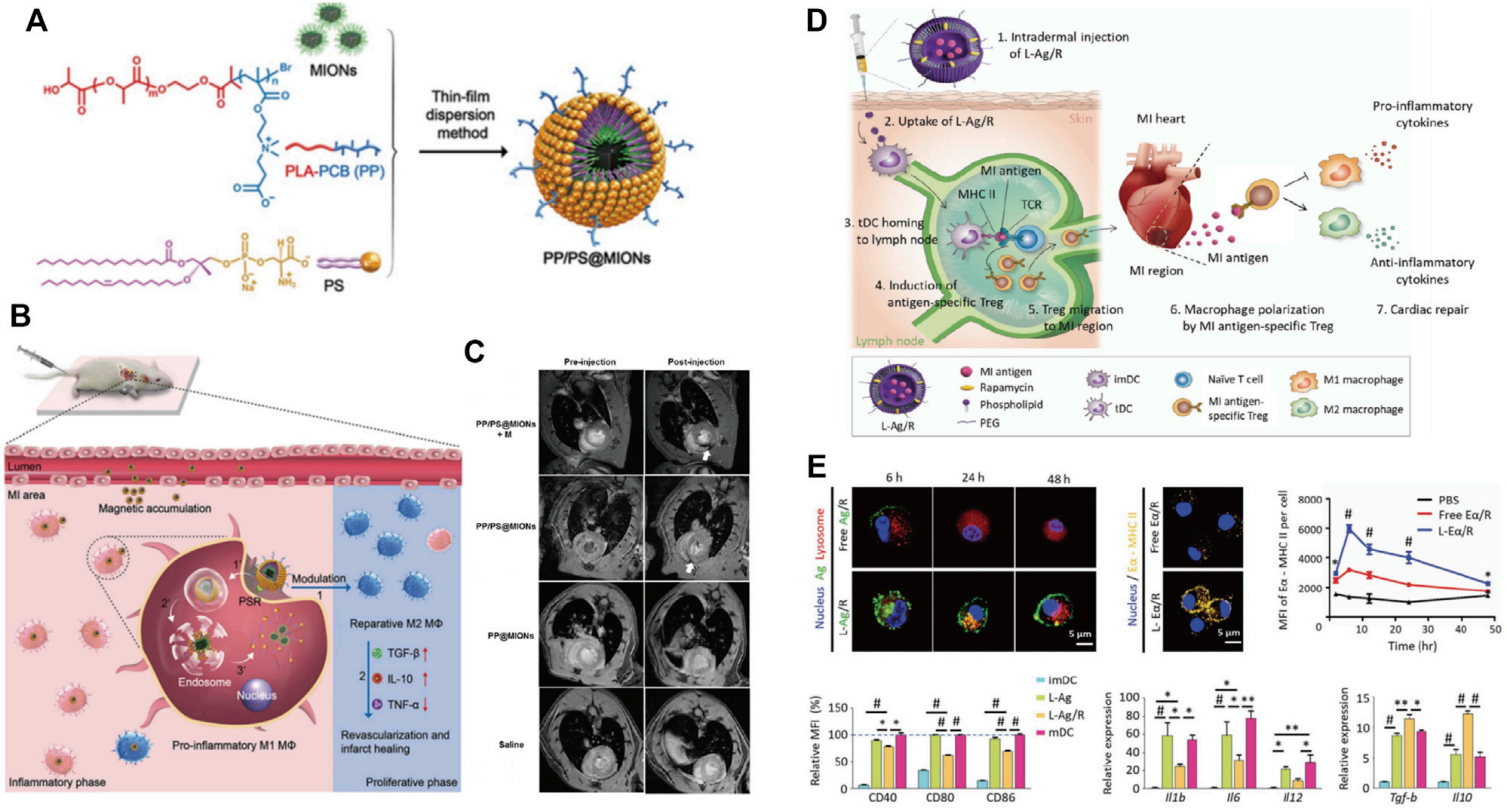
Nanomaterials use immune-modulation strategy to treat MI. **(A)** Chemical construction of PP/PS@MIONs nanotheranostic system by the thin-film dispersion method. **(B)** Schematic illustration for PP/PS@MIONs in MI. PP/PS@MIONs were accumulated in the MI area due to magnetic targeting and PS targeting and promoted the differentiation of the pro-inflammatory macrophages (M1) into the reparative macrophages (M2). **(C)** Representative MR images of hearts before and 24 h after *i. v*. administration of PP/PS@MIONs and PP@MIONs with or without a magnet. Adapted with permission from Chen et al., 2017. **(D)** Schematic illustration of MI treatment using liposomal nanoparticles loaded with MI-associated antigens and rapamycin (L-Ag/R). **(E)** The delivery of L-Ag/R promoted the antigen presentation efficiency of DCs, inhibited the expressions of co-stimulatory surface molecules and pro-inflammatory cytokines expression in DCs, and increased the expressions of anti-inflammatory cytokines in DCs. Adapted with permission from Kwon et al., 2021.

As the phagocytes, macrophages enable the endocytosis of apoptotic cells and differentiate from the pro-inflammatory macrophages (M1) to the reparative phenotype (M2), resulting in an anti-inflammatory response. This process depends on the specific recognition between the externalized phosphatidylserine (PS) on membranes of apoptotic cells and the PS receptors (PSR) on the macrophages’ surface. Hence, the presentation of PS in nanoparticles benefits suppressing the inflammation by mimicking the interface chemistry of apoptotic cells, leading to the targeting and polarization of macrophages. Chen et al*.* prepared PS in a zwitterionic biodegradable copolymer poly (lactide)-polycarboxybetaine (PP) and encapsulated magnetic iron oxide nanocubes (MIONs) within it (Figure 3A) (Chen et al., 2017). Under the dual effects of magnetic induction and PS targeting, PP/PS@MIONs were accumulated in macrophages within the infarcted tissues and then converted the pro-inflammatory macrophages (M1) to a reparative phenotype (M2) after the PS and PSR engagement, thus regulating the anti-inflammatory responses in the cardiac microenvironment and improving the MI healing (Figure 3B). On the other hand, the release of MIONs enhanced the theragnostic signal of MRI, realizing accurate visualization of MI at the early stage (Figure 3C).

In addition to macrophages, the antigen-specific immune tolerance and the macrophage polarization can be modulated in the infarct region by direct targeting the immune cells such as DCs. To induce antigen-specific immune tolerance in MI tissue, Kwon et al*.* designed PEGylated 1,2-dioleoyl-3-trimethylammonium-propane (DOTAP) liposome nanoparticles by entrapping lysate of MI tissue as the MI antigens and rapamycin (L-Ag/R) as the tolerogenic dendritic cells (tDC) inducer (Figure 3D) (Kwon et al., 2021). Notably, it is necessary to incorporate multiple antigen types since the use of myosin or troponin failed to induce immune tolerance. The obtained nano-complex named L-Ag/R nanoparticles that were 200 nm in size facilitated the uptake by DCs, which then established a robust and prolonged antigen presentation of DCs through MHC-II. The co-delivery of rapamycin further induced a tolerogenic phenotype in DCs by downregulation of co-stimulatory molecules and pro-inflammatory cytokines, upregulation of immuno-suppressive cytokines, and suppression of CD4^+^ T cell proliferation (Figure 3E). Moreover, the antigen presentation efficiency and tDC generation resulted in the systemic generation of Tregs in the inguinal lymph node, which polarized pro-inflammatory M1 macrophage to a reparative M2 phenotype, leading to reduced post-MI adverse remodeling and cardiac dysfunction.

### 2.3 Antioxidants and Antiapoptosis Strategy

In the early stage of MI, a continuous ischemic microenvironment leads to mitochondrial oxidative phosphorylation uncoupling and imbalances of calcium homeostasis, which cause the massive generation of reactive oxygen species. The overloaded oxidative free radicals within the heart aggravate the oxidative stress injury of the heart. Combined with the local ATP deficiency, apoptosis and necrosis of cardiomyocytes start to occur quickly within 24 h after MI (Cabac-Pogorevici et al., 2020). Due to the absent self-regenerative ability of cardiomyocytes, early prevention of apoptosis becomes a critical strategy for alleviating myocardial injury and promoting cardiac repair.

Applications of antioxidative nanomaterials in the early stage of MI have been proved an effective strategy to reduce cardiac injury caused by MI. Notably, some nanomaterials elicit specific oxidative damage resistance properties due to their own unique structures. For example, Zhang et al*.* reported that tetrahedral DNA nanoparticles displayed great antioxidative and anti-inflammatory potentials in myocardial ischemia-reperfusion injury (MRI) (Zhang M et al., 2019). The application of tetrahedral DNA nanoparticles significantly inhibited the overproduction of ROS and decreased the oxidative damage and apoptosis of MIRI. In addition, metal nanoparticles also show an attractive ability to reduce oxidative injury. They can be cooperated with other protein structures to construct functional nanocomposites. For an instant, Zhang et al*.* constructed a new artificial mixed nano-enzyme, with the metal nanoparticles as the active center and ferritin heavy chain protein as the scaffold (Zhang et al., 2021). This artificial nano-enzyme with activities of superoxide dismutase and catalase could target mitochondria to inhibit the superoxide of mitochondria and reduce the oxidative stress caused by myocardial ischemia-reperfusion injury. Therefore, this kind of nano-enzyme with intrinsic enzyme activity is considered one of the effective potential nanomaterials for inhibiting oxidative damages.

**FIGURE 4:**
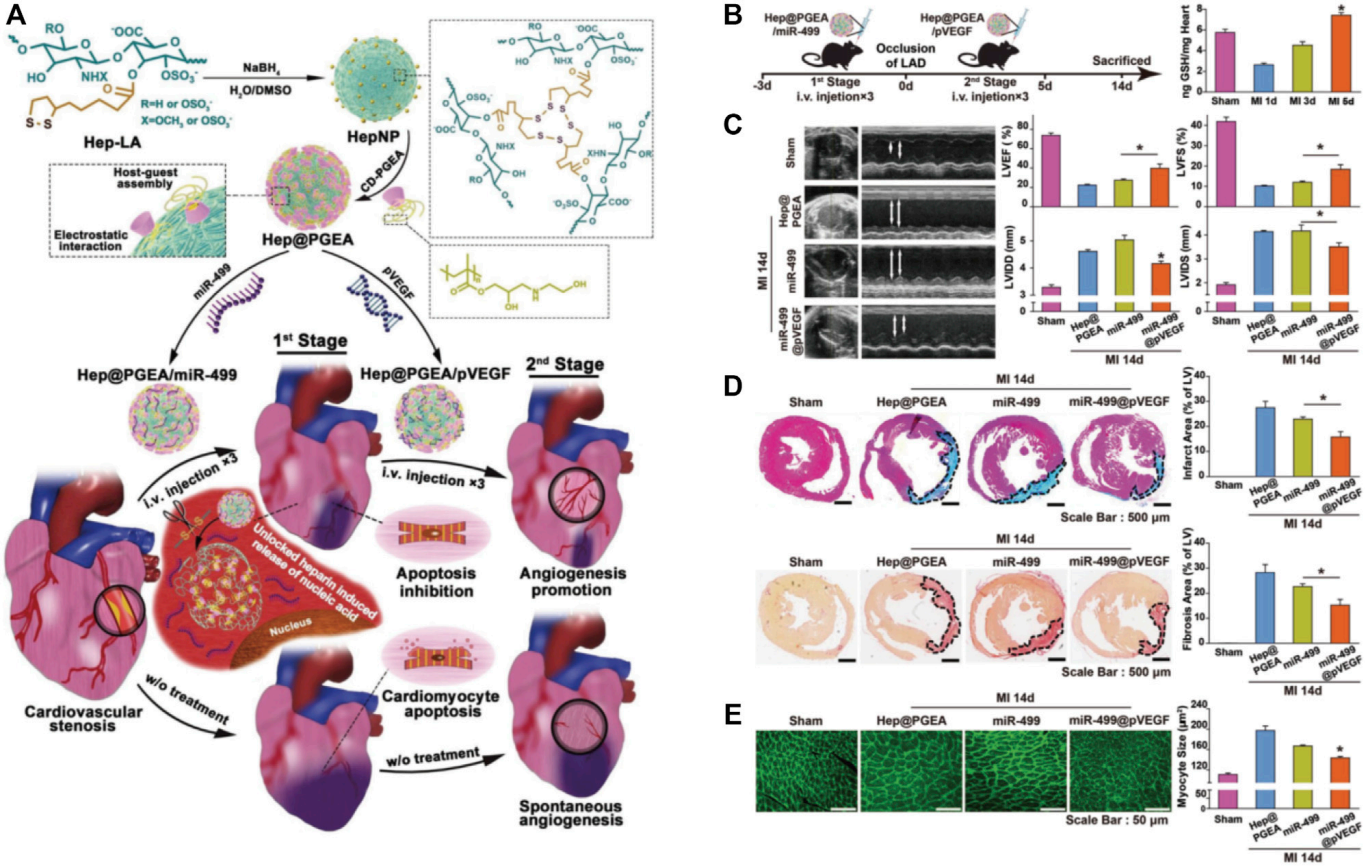
The application of nanomaterials for the delivery of miRNA and pDNA to inhibit the cardiomyocytes apoptosis in MI. **(A)** Schematic illustration of the construction of unlockable heparin core-shell nanocomplexes (Hep@PGEA) and their applications in the delivery of miRNA and pDNA for the treatment of MI. **(B)** The time axis of this study and the evaluation of GSH amount in mouse hearts at different time points after MI. **(C)** Hep@PGEA preserved the cardiac function of mice after MI evaluated by M-mode echocardiograms. **(D)** The delivery of Hep@PGEA reduced the infarct areas and fibrosis areas reflected by quantification of Masson trichrome and Red Sirius staining. **(E)** Hep@PGEA inhibited the cardiomyocytes hypertrophy reflected by the WGA staining. Adapted with permission from Nie et al., 2018.

However, the strategies to inhibit cardiomyocyte apoptosis by nanotechnology are mainly mediated by applying nanomaterials as carriers to deliver active therapeutic agents, such as micro-RNAs, growth factors, and exosomes. Nie et al*.* investigated the nucleic acid-based therapy for MI and designed an unlockable core-shell nano-complex (Hep@PEGA) for the incorporation of miR-499 (an effective cardiomyocytes apoptosis inhibitor) and plasmid encoding vascular endothelial growth (pVEGF) (Figures 4A,B) (Nie et al., 2018). The nucleic acid-containing nano-complex effectively inhibited the apoptosis induced by MI and restored cardiac function (Figure 4C). Furthermore, the infarct areas, fibrosis areas, and cardiomyocytes hypertrophy areas were also significantly inhibited by Hep@PEGA (Figures 4D,E). Besides, nanoparticles are also designed for the delivery of growth factors to inhibit the apoptosis of cardiomyocytes. Chang et al*.* designed an insulin-like growth factor-1 (IGF-1) loaded polylactic co-glycolic acid (PLGA) nanoparticles to release IGF-1 in the myocardium after intramyocardial injection. The composite nanoparticles exert their effect in inhibiting myocardial apoptosis by activating AKT phosphorylation (Chang et al., 2013). Lastly, exosomes, single-membrane, secreted organelles of ∼30 to ∼200 nm in diameter also show promising potential in inhibiting apoptosis and promoting post-MI repair (Xiong et al., 2021). Gao et al*.* utilized exosomes secreted by induced pluripotent stem cells (iPSCs) to treat MI, which significantly reduced cell apoptosis and reduced the incidence of arrhythmia and other complications (Gao et al., 2020). In addition, exosomes can also be applied as useful vehicles for the delivery of those antiapoptosis substances. Yao et al*.* constructed a self-assembled stem cell membrane-camouflaged exosome-mimicking nano-complex with a mesoporous core for miR-21 (Yao et al., 2020). This nano-complex provides cloaking from clearance of the mononuclear phagocytic system (MPS) and helps the miR-21 to locate in the infarcted area. The slow release of miR-21 in the infarcted site then exerted these antiapoptosis effects and protected against the cardiac repair after MI.

### 2.4 Nanomaterials-Mediated Stem Cell Therapy

Several studies have revealed that delivering stem cells or progenitor cells to the infarcted areas could promote cardiac function and inhibit the adverse remodeling after MI (Guo et al., 2020; Prasad et al., 2020). However, low transplantation and survival rates have become major problems for stem cell therapy in MI treatment. Besides, arrhythmia, adverse immune rejection, and tumor risk caused by stem cell transplantations are the other problems needing to be addressed (Ong and Wu, 2015). Fortunately, nanomaterials have provided solutions to these problems by improving the cell survival environment, promoting cell proliferation, and regulating the differentiation of transplanted cells in the ischemic areas (Chistiakov et al., 2018). PLGA nanoparticles loaded with simvastatin promoted the differentiation of adipose-derived stem cells into smooth muscle and endothelial cells, thus playing a protective role in MI models (Yokoyama et al., 2019). Zhang et al. labeled endothelial progenitor cells with magnetic iron oxide nanoparticles coated with silica to form magnetic endothelial progenitor cells (Zhang B. F. et al., 2019). Under the guidance of an external magnetic field, magnetized endothelial progenitor cells could be transferred to the infarcted areas of the hearts after tail vein injection. Due to the significant promotion of the aggregation of endothelial progenitor cells at the edge of the infarcted regions, the therapeutic effect on MI was improved. More interestingly, Cheng et al*.* combined iron oxide nanoparticles with two types of antibodies, CD45 antibody, a bone marrow stem cell surface marker, and myosin light chain (MLC) antibody, a damaged myocardium marker (Cheng et al., 2014). The synthesis of nanoparticles acted as the magnetic cell adapter under the external magnetic effects and promoted the interaction between endogenous bone marrow stem cells and damaged cardiomyocytes. This novel solution overcomes the inefficient interaction problem involving the stem cells for cardiac repair, the injured cardiomyocytes, and the therapeutic efficiency of the stem cells for MI.

**FIGURE 5:**
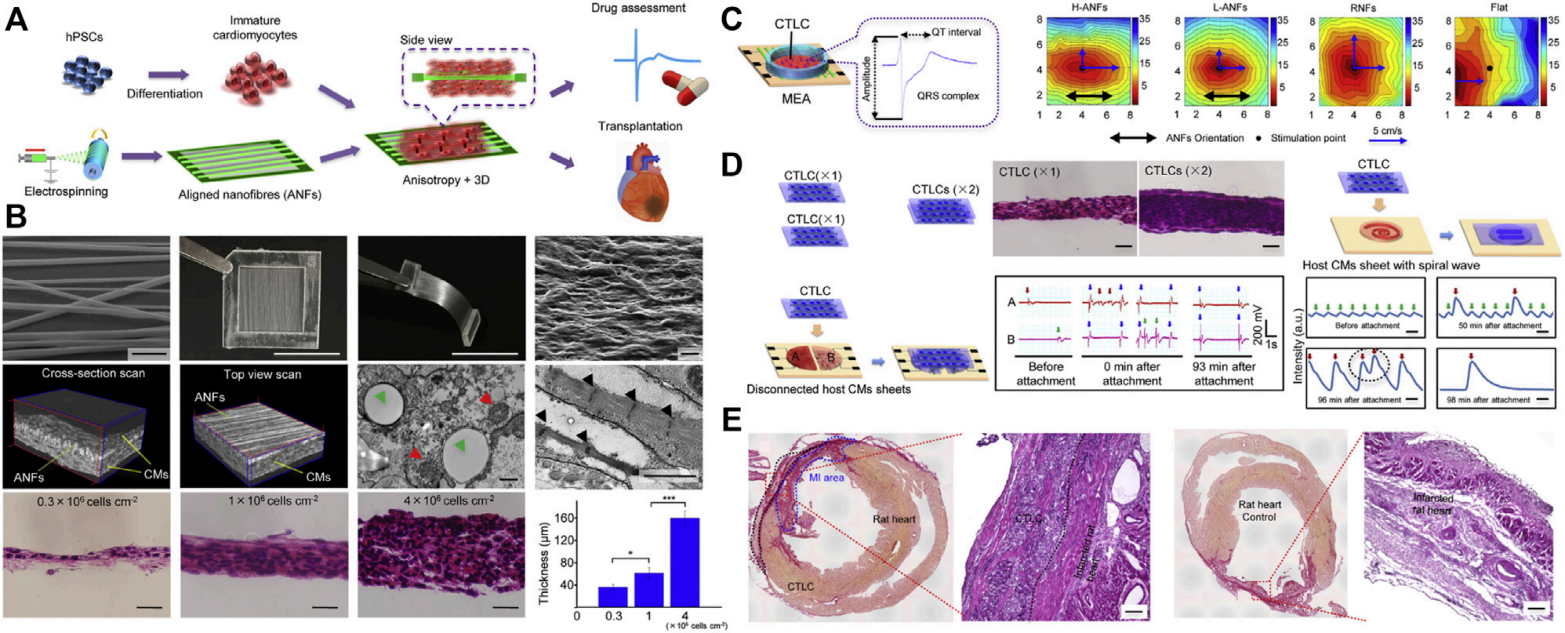
The strategy of nanomaterials assistant stem cell therapy for MI. **(A)** Schematic illustration of the poly (lactic-co-glycolic acid) (PLGA) fabricated aligned nanofibers (ANFs) for the high-quality cardiac tissue-like constructs (CTLCs) differentiated from human induced pluripotent stem cells (hiPSCs). **(B)** The construction of PLGA-mediated ANFs and their effects on the 3D cardiac tissue-like constructs (CTLCs). **(C)** Schematic representation of CTLCs on the microelectrode array (MEA) and a representative electrogram of the field potential (FP). Activation maps showing the propagation of stimulated contractility on day 6. **(D)** CTLCs synchronize disconnected cardiomyocyte tissues and suppress re-entrant arrhythmia within scarred cardiomyocyte sheets. **(E)** Histological sections of CTLCs on the MI tissues cultured by aligned nanofibers and acellular nanofibers, respectively. Adapted with permission from Li et al., 2017.

Besides, induced pluripotent stem cells (iPSCs) therapy is considered an effective approach to promoting myocardial regeneration. iPSCs have the potential to differentiate into cardiomyocytes to replace the damaged cardiomyocytes. However, there are still some differences in morphological structure and physiological function between iPSCs differentiated cardiomyocytes and mature cardiomyocytes. The application of nanomaterials is expected to break through this bottleneck. Li et al*.* cultured hiPSC differentiated cardiomyocytes on low thickness aligned nanofibers synthesized from degradable PLGA polymers and obtained more mature cardiac tissue-like constructs (CTLCs) (Figures 5A,B) (Li et al., 2017). The CTLCs showed great features in the propagation of stimulated contractility (Figure 5C). Besides, the obtained CTLCs repaired the disconnected cardiomyocyte tissues and suppressed re-entrant arrhythmia within the scarred region (Figure 5D), thus protecting the hearts against the MI (Figure 5E).

### 2.5 Nanomaterials-Mediated Cardiac Tissue Engineering

Cardiac engineering biomaterials aim to create supportive biological materials for repairing infarcted hearts, which are mainly divided into cardiac patches and scaffolds composed of bioactive hydrogel materials and nanofibers. In addition to serving as a carrier for therapeutic drugs, their specific microstructures provide appropriate mechanical elasticity and support for the hearts through minimally invasive local injection (Izadifar et al., 2018). However, the unmodified injectable hydrogels or nanofibers do not provide effective electrical conduction of cardiomyocytes at the injection site and tend to rapidly degrade due to the continuous dynamic motion of the hearts. To fully exert the protective effects of these engineering biomaterials, improving their electrical conductivity, mechanical strength, and structural integrity are crucial pivots for modifications. Nanotechnology can improve the physical properties and electrical conductivity of biomaterials, which help to accommodate cardiomyocytes and promote their synchronized beating behaviors within the biomaterials. The combination of nanotechnology with biomaterials engineering could take both advantages and achieve better therapeutic effects.

Nano-gel prepared from polymer nanomaterials is one of the most commonly used tissue engineering biomaterials (McClements, 2017). The most common application of nanomaterials combined with the myocardial patch is by introducing nanomaterials with good conductivity. The conductivity and mechanical strength of hydrogels can be enhanced by the application of gold nanorod (GNR) into the hydrogel matrix of Methacrylate gelatin (GelMA). As compared with pure GelMA hydrogel, the GelMA-GNR hydrogel showed improved retention of cardiac cells, cell viability, metabolic activity, cell-matrix interaction, and synchronous beating of cardiac tissue, indicating that functional heart patches with higher electrical conductivity and mechanical strength can be developed by using nanotechnology (Navaei et al., 2016). A similar design for cardiac protection was verified by Chen et al*.* (Chen et al., 2021). They designed an injectable hydrogel comprising phenylboronic acid hyperbranched macromer, within which gold nanorods and Astragaloside IV (AST NPs) nano drug were encapsulated. The composite hydrogel displayed great properties of electrical stimulation mediated by the gold nanorods and protected against the adverse left ventricular remodeling by the slow release of AST NPs.

**FIGURE 6:**
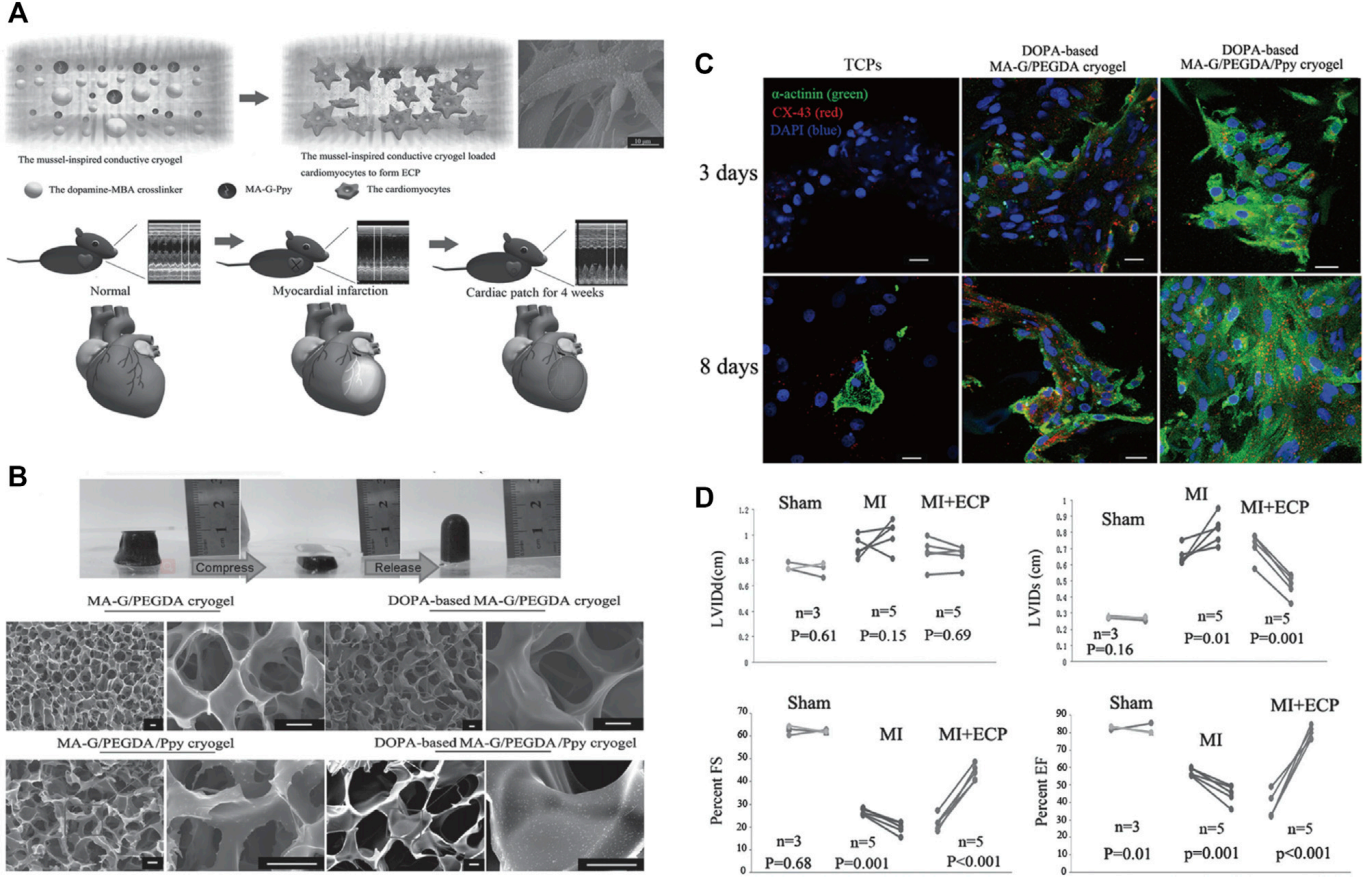
Mussel-inspired conductive cryogel as a promising strategy for the restoration of infarcted myocardium. **(A)** Schematic illustration of the mussel-inspired conductive cryogel for engineered cardiac tissue patch in rat MI models. **(B)** Characterization and ultrastructure of the DOPA-based Ppy PEG-gelatin cryogel. **(C)** The DOPA-based MA-G/PEGDA/Ppy cryogel showed higher protein expressions of α-actinin and CX-43 in the cardiomyocytes presented by immunofluorescent staining. **(D)** The DOPA-based MA-G/PEGDA/Ppy cryogel significantly improved the cardiac functions of the mice after MI. Adapted with permission from Wang et al., 2016.

In addition to the hydrogels, nanomaterials can also improve the biological properties of cardiac patches. In a study by Wang et al*.*, they constructed an engineered cardiac patch by integrating polypyrrole (Ppy) nanoparticles, gelatin-methyacrylate, and poly (ethylene glycol) diacrylate (PEGDA) into the cryogel using mussel-inspired dopamine (DOPA) as the crosslinker (Figure 6A) (Wang et al., 2016). The introduction of Ppy nanoparticles and DOPA crosslinker optimized the mechanical and superelastic properties of the DOPA-based cryogel (Figure 6B). The novel cryogenic myocardial patches showed good performances for the functionalization of cardiomyocytes, evidenced by the increased expressions of α-actinin and CX43 (Figure 6C). So the implantation of cryogenic myocardial patches significantly reduced myocardial infarction areas in rats and improved cardiac function after myocardial infarction (Figure 6D). Based on this novel finding, this research group further improved the conductivity of the PEGDA-GelMA cryogel by embedding MXene Ti_2_C (Ye et al., 2020). The newly designed Ti_2_C-8-cryogel showed suitable conductivity and mechanical properties and served as a good candidate for the treatment of MI.

Nowadays, nanomaterials combined with engineering 3D printing platforms to improve the acceptability of implants and reconstruct infarction hearts is a very promising research direction. Mehrotra et al*.* used a conductive bioink containing carbon nanotubes (CNTs) to bioprint functional 3D vascularized anisotropic heart structures (Mehrotra et al., 2021). It was found that in addition to upregulating mature cardiac biomarkers, sarcomere formation, and beat rate, CNTs also promoted cardiomyocyte activity through immunofluorescence staining, polymerase chain reaction-based gene expression studies, and electrophysiological studies.

## 3 Nanomaterials-Mediated Diagnosis Strategies for MI

Prevention is better than cure, and thus there is a growing need for more sensitive diagnostic tools for MI. Since 85% of heart damage occurs in the first 2 hours after a heart attack, early diagnosis and classification of patients based on prognosis can significantly reduce the complications of MI, thus saving lives and reducing mortality. Current common clinical MI diagnosis methods include electrocardiogram, physical examination, imaging, and MI-related biomarkers detection (Stengaard et al., 2016). However, each of these methods has some limitations and disadvantages, including expensive, poor sensitivity and specificity, the need for advanced equipment, and a trained operator. These limitations are in contrast to the necessity of rapid diagnosis of MI, which is key to managing symptoms, reducing ischemic progression, and controlling disease after MI. The physicochemical and optical properties of nanomaterials are considered productive contrast agents with high-resolution biological imaging modes, making disease tracking more feasible.

### 3.1 Nanomaterials-Based Immunoassay

Infarct healing and cardiac remodeling after MI involve a complex series of interrelated cellular and molecular events, which are regarded as indicators of the risk and progression of MI (Liehn et al., 2011). They can be detected and quantified through various immuno-assays based on antigen-antibody immunoaffinity, such as electrochemiluminescence (ECL), photoelectrochemistry (PEC), surface enhanced raman scattering (SERS), surface plasmon resonance (SPR), and enzyme-linked immunosorbent assay (ELISA). Measuring the expression levels of specific biomarkers with immunoassays has the advantages of high sensitivity, rapid, cheap, and non-invasive for the prediction and diagnosis of MI. Although relatively satisfactory results have been obtained from previous methods, high sensitivity and accuracy in testing results are still pursued. In addition to the high binding efficiency of cardiac targets, nanomaterials can reduce non-specific binding sites and offer excellent signal amplification ability (Hao et al., 2021; Low et al., 2020; He et al., 2022). Hence, combining nanomaterials with immunoassays may serve as a solution for early-stage MI diagnosis. The summary of nanomaterials-based immunoassay for MI diagnosis is provided in Table 2.

**TABLE 2:** Summary of nanomaterials-based immunoassay for MI diagnosis.

Cardiac biomarker	Immunoassay	Nanomaterials	Detection limit	References
Mb	ECL	AuNPs	34.6 ng/ml	Adeel et al. (2019)
	ECL	QDs	0.0492 ng/ml	He et al. (2022)
	SPR	RGO	4 pg/ml	Singh et al. (2020)
cTnI	ECL	QDs	0.0005 ng/ml	Yola and Atar, (2019)
	ECL	QDs	0.0184 ng/ml	He et al. (2022)
	PEC	ZnO	0.003 pg/ml	Liao et al. (2021)
CK-MB	ECL	AuNPs	0.62 pg/ml	Wang et al. (2020)
BNP	ECL	AuNPs	0.11 pg/ml	Dong et al. (2019)
GSH	PEC	CDs	6.2 nmol/L	Li et al. (2018)

Mb, myoglobin; cTnI, Troponin-I; CK-MB, creatine kinase-MB; BNP, B-type natriuretic peptide; GSH, glutathione; SPR, surface plasmon resonance; ECL, electrochemiluminescence; PEC, photoelectrochemistry; AuNPs, gold nanoparticles; QDs, quantum dots; RGO, reduced graphene oxide.

**FIGURE 7:**
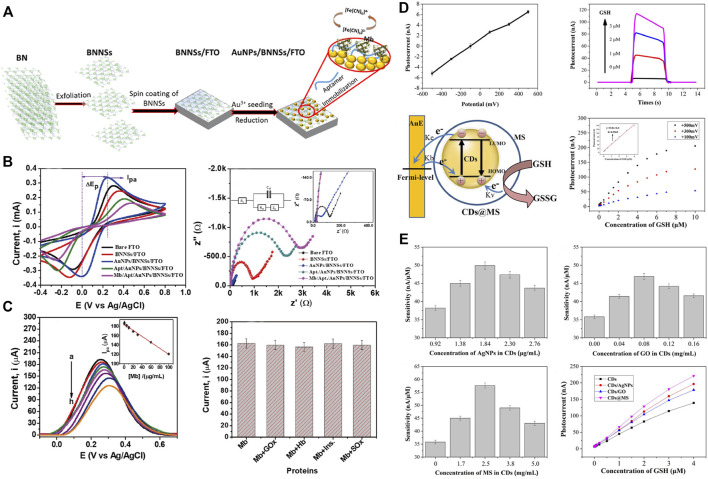
Nano-biosensor based on electrochemical or photoelectrochemical for the detection of MI-related indicators. **(A)** Schematic illustration for the fabrication processes of the AuNPs/BNNSs nanosheets and the detection of myoglobin (Mb). **(B)** Electrochemical characterization of AuNPs/BNNSs nanosheets. **(C)** Detection of Mb and interference studies, stability, reproducibility, and real-sample analyses of AuNPs/BNNSs nanosheets. Adapted with permission from Adeel et al., 2019. **(D)** The mechanism of the carbon dots nano-biosensor based on photoelectrochemical for the detection of glutathione (GSH). **(E)** Sensitivity and photocurrents the carbon dots-based biosensors introducing silver nanoparticles, graphene oxide, and mesoporous silica. Adapted with permission from Li et al., 2018.

It is generally known that myoglobin (Mb) is a crucial cardiac biomarker for early detection and diagnosis of myocardial infarction as it is released into the bloodstream once there is an incidence of cardiac muscle injury (Garg et al., 2017). Hence, monitoring the myoglobin level could facilitate the early detection and appropriate therapeutic measures for MI. In this regard, Adeel et al*.* designed a novel electrochemical aptasensor based on gold nanoparticles decorated on boron nitride nanosheets (AuNPs/BNNSs) for the sensitive and selective detection of Mb (Figure 7A) (Adeel et al., 2019). The results reveal that this nanoparticle sensor detected the electrochemical changes of myoglobin, which is considered an excellent sensing platform for the rapid detection of MI (Figures 7B,C). The nano-biosensors for MI detection based on electrochemical impedance are proved to be suited for point-of-care applications owing to their ease of miniaturization and low cost. Aside from Mb, various other antibodies against biomarkers for MI, including cardiac Troponin-I (cTnI) (Cutlip et al., 2007), creatine kinase-MB (CK-MB) (Apple and Murakami, 2005), B-type natriuretic peptide (BNP) (Thygesen et al., 2018), and glutathione (GSH) (Pastore and Piemonte, 2013), have been used to create single site-specific polyaniline nanowire biosensors. In one of those studies, a novel imprinted biosensor approach based on boron nitride quantum dots (BNQDs) was presented for cTnI detection in plasma samples. It was found that the prepared biosensor has high stability, repeatability, and reproducibility in the detection of cTnI (Yola and Atar, 2019).

In addition to the nanobiosensors that are developed based on electrochemical methods, the fluorescence immunoassay (Piperigkou et al., 2016), photoelectrochemical immunoassay (Zhao et al., 2015), and surface-enhanced raman scattering immunoassay (Driskell et al., 2005) have also attracted broad attention owing to their high sensitivity. PEC sensors based on carbon dots (CDs) were developed for ultrasensitive detection of GSH levels, which correlates with the pathophysiology of MI. Silver nanoparticles, graphene oxide, and mesoporous silica were introduced to improve the sensitivity of carbon dots-based PEC sensors, among which the carbon points related to mesoporous silica performed best (Figures 7D,E). Thus, this concept could facilitate the early detection of MI and therefore provide necessary inputs for further therapeutics (Li et al., 2018).

### 3.2 Nanomaterials-Enhanced Cardiac Imaging

As a common clinical diagnostic tool for CVDs, cardiac imaging can reveal the individual biological information of the heart (Cheng and Pu, 2021). Common cardiac imaging approaches include magnetic resonance imaging (MRI), photoacoustic tomography (PAT), CT, positron emission tomography (PET), and single-photon emission computed tomography (SPECT) (Tamis-Holland et al., 2019). Due to the good bioavailability and versatility of nanomaterials, the accuracy and specificity of clinical cardiac imaging applications can be elevated by improving resolution and amplifying the signal. Meanwhile, owing to the mobility of nanoparticles in the internal and external vascular system, high surface area to volume ratio, and imaging functionality, nanoparticles are less restricted in circulation in the human body. When applied to cardiac imaging, nanomaterials can be used as contrast agents to generate functional imaging carriers, thus significantly improving diagnostic efficiency (Weissleder et al., 2014; Zhang et al., 2018).

**FIGURE 8:**
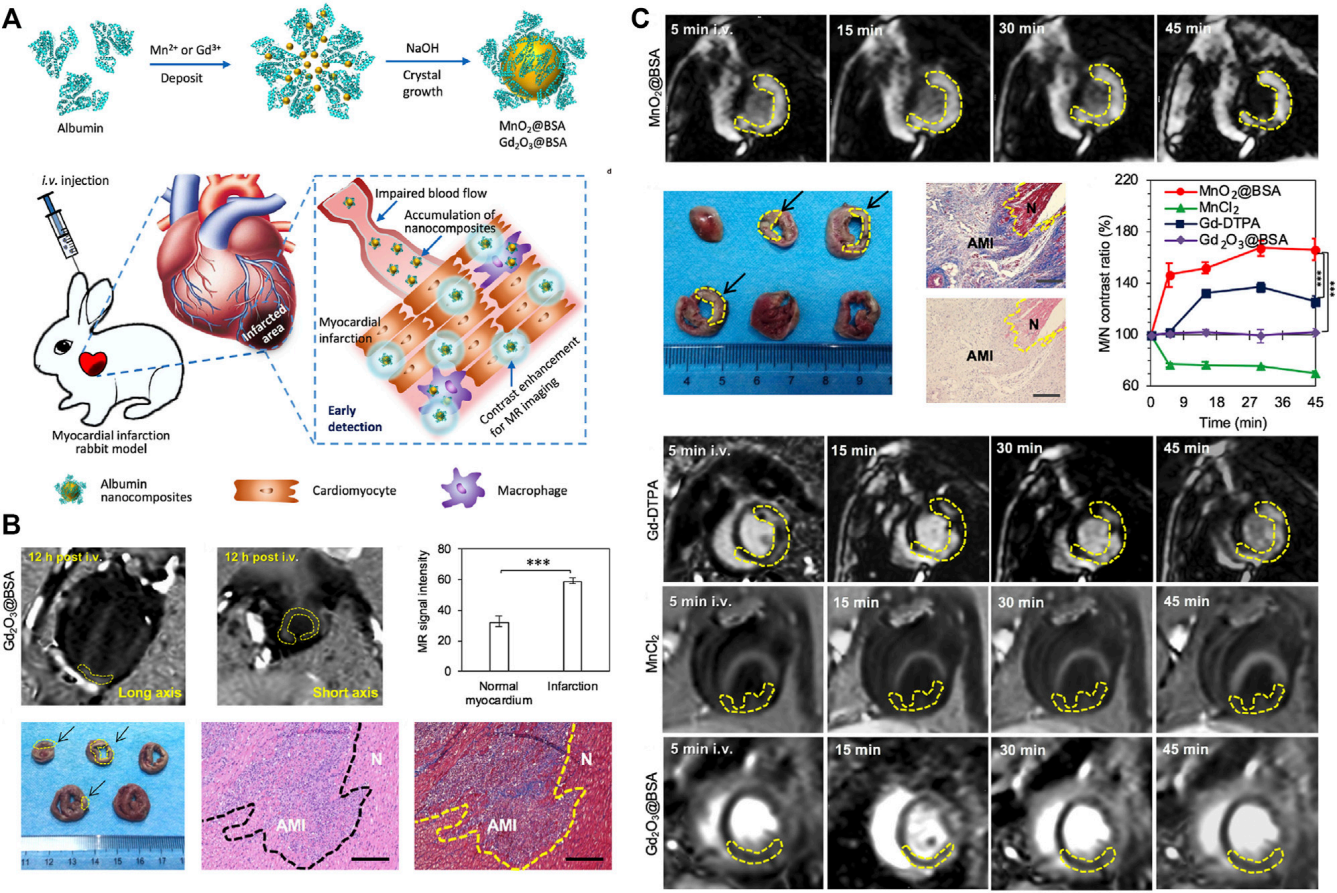
MnO_2_/Gd_2_O_3_ nanocomposites used as a contrasting agent for MRI bioimaging modalities for sensitive detection of MI. **(A)** Schematic illustration of MnO_2_@BSA and Gd_2_O_3_@BSA nanocomposites for MR imaging of MI in rabbit models, the MnO_2_@BSA nanocomposites could be accumulated in MI regions and response to the low pH to liberate Mn^2+^ to achieve specific contrast enhancement for MR imaging of MI. **(B)** MR imaging of acute myocardium infarction in rabbits contrasted by Gd_2_O_3_@BSA nanocomposites. **(C)** MR imaging of acute myocardium infarction in rabbits contrasted by MnO_2_@BSA nanocomposites. Adapted with permission from Wang et al., 2020.

Cardiac MRI, which provides a highly accurate view of the cardiac anatomy, is recognized as the gold standard for the non-invasive evaluation of MI (Ibanez et al., 2019). Nanoparticles could be ideal contrast agents for MRI. The density and location of nanoparticles in MRI can be modulated by the integration of targeting moieties to their interface. Super-paramagnetic nanoparticles, like iron oxide nanoparticles and predominately magnetite (Fe_2_O_3_/Fe_3_O_4_), can improve the sensitivity of MRI by providing dark contrast to enhance the signal. Hu et al*.* constructed an external magnetic field-responsive iron oxide nanocubes as a nanoplatform for MRI monitoring and selective targeting of macrophages in external magnetic field-induced infarcted tissues. The prepared iron oxide nanocubes, with good biocompatibility and magnetic target ability to the heart, can perform qualitative and quantitative MRI examinations of MI (Hu et al., 2016). Similarly, manganese oxide nanoparticles were engineered and directed to use as a contrasting agent for MRI bioimaging modalities for early detection of MI (Figure 8A). The pH-sensitive albumin nanocomposites with MnO_2_ motifs (MnO_2_@BSA) have been engineered for MRI of MI. The results showed that MnO_2_@BSA had a high accumulation in the area of MI and was rapidly metabolized from the body after systemic injection. With high contrast enhancement, it could accurately image the MI in the rabbit model, showing excellent diagnostic performance (Figures 8B,C) (Wang et al., 2020).

As a mature medical imaging technique, ultrasound (US) imaging is characterized by convenient, fast, and low cost (Lin et al., 2020), and therefore is well suited for monitoring cardiac movement changes and identifying CVD (Kiessling et al., 2014). Combining the unique advantages of US imaging with nanomaterials and attaching specific molecular markers to the surface of nano materials enables these nano scale contrast agents to target MI-specific antigens or antibodies. Zhou et al*.* designed an US-based multifunctional CNA35-labeled perfluoropentane nanoparticles (CNA35-PFP NPs) and injected them intravenously into a rabbit model of MI (Zhou et al., 2019). It was found that CNA35-PFP NPs can effectively cross the interstitial space of endothelial cells and adhere to the surface of fibroblasts. Importantly, these CNA35-PFP NPs can be converted from liquid to gaseous microbubbles after low intensity focused ultrasound irradiates on the myocardium, further significantly enhancing the ultrasound contrast of the fibrosis region and facilitating the detection of ultrasound diagnostic imaging. The non-invasive, economical, real-time imaging technique provides a certain research basis for the clinical application of nano-based US imaging.

## 4 Conclusion and Future Prospect

Recently, innovation and development based on nanomaterials applications in the biomedical field have led to encouraging results in the therapeutic and diagnostic of MI. In this article, we systematically reviewed the unique advantages of nanomaterials in improving the targeting, exerting their cardiac protective effects, and prompting the applications of nanomaterials in therapy and diagnosis during MI. Summarizing these recent research focuses will provide clinicians with insight into the therapeutic and diagnostic strategies of MI based on nanomaterials. Nevertheless, there are still some challenges needed to be addressed for the applications of nanomaterials in clinical practice. Firstly, most studies have focused on the beneficial effects of nanomaterials, whereas systematic toxicity and biocompatibility of nanomaterials are rarely reported. The off-target effects of nanomaterials used as the drug delivery system lead to drug accumulation in other irrelevant target organs, thus aggravating the toxic side. Besides, efforts should be made to develop efficient and stable approaches for the production of nanomaterials and improve their encapsulation efficiency in the future. Furthermore, more research is required to develop nano systems that can respond to the optical, electrical, or magnetic signals, which can be used by cells to stimulate the positive signals and exemplified by a dual-responsive nano system (Daniel et al., 2016). Secondly, the cardiac repair is complex and involves many processes, including apoptosis, oxidative stress, angiogenesis, inflammatory infiltration, and fibrosis. The strategies based on these critical pathophysiological changes are instrumental in improving the targeting of the treatment. However, they can still suffer from off-target accumulation when those pathophysiological changes are not only existed in the injured hearts, especially for the patients with cancer or some immune diseases. So future efforts should be more paid on the discovery of MI-specific targets or the development of non-invasive cardiac *in situ* techniques. Thirdly, the therapeutic efficiency of stem cell therapy has been greatly improved by nanomaterials, including the improvement of targeting and maturity of differentiation. Nevertheless, the risks of arrhythmia, adverse immune rejection, and tumor risk have not been completely eliminated. Therefore, novel strategies are required to be explored to preserve the curative effects of stem cell therapy while avoiding their disadvantages, such as stem cell-derived extracellular vesicles. Although the features of electrical conduction and mechanism have been greatly improved, it remains to be solved how to further improve the survival of the injured cardiomyocytes in the early stage and develop the non-invasive delivery approach. Overall, future research should focus on these areas to translate nanomaterials-based MI treatment strategies into practical and effective clinical applications.
